# Bone Loss and TLR4 Signals Contribute Independently to B Lineage Aging

**DOI:** 10.1111/acel.70267

**Published:** 2025-10-11

**Authors:** Erin Baker, Encarnacion Montecino‐Rodriguez, Shili Xu, Sotirios Tetradis, Adrien Rouault, Oscar I. Estrada, Kenneth Dorshkind

**Affiliations:** ^1^ Departments of Pathology and Laboratory Medicine UCLA School of Dentistry Los Angeles California USA; ^2^ Molecular and Medical Pharmacology, David Geffen School of Medicine at UCLA UCLA School of Dentistry Los Angeles California USA; ^3^ Division of Diagnostic and Surgical Sciences UCLA School of Dentistry Los Angeles California USA

**Keywords:** aging, B cells, bone marrow, environment

## Abstract

B cell development declines with age, but how structural changes in the marrow environment contribute to that process is incompletely understood. Multiplexed volumetric imaging revealed that B lineage cells were enriched near bone, and trabecular bone in particular, in young mice. However, B cell progenitors were depleted from these regions in strains of old mice that exhibited senile osteoporosis. In striking contrast, the age‐related decline of B lymphopoiesis was attenuated in mice in which bone mass was maintained over the lifespan and could be completely abrogated by concomitantly blocking TLR4 signaling. In addition to demonstrating that developing B lineage cells are not randomly distributed in the marrow, these results indicate that the age‐related decline in B lymphopoiesis is influenced by the loss of salutary and not just an increase in inhibitory signals.

## Graphical abstract

The prevailing view is that the age‐related decline of B lymphopoiesis is due to increased inhibitory signals in the marrow environment. Our data show that the loss of salutary ones, such as those from bone, primarily drives the loss of B cell progenitors with age.

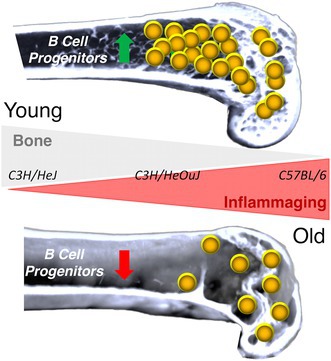

## 1 Introduction

The bone marrow is the site of B cell development after birth, and that process is robust in young individuals. However, there is a consensus that B lymphopoiesis is attenuated with age resulting in a significant decrease in the number of cells at all stages of development. In this regard, the number of common lymphoid progenitors (CLPs) (Min et al. 2006), pro‐B (Miller and Allman 2003; Min et al. 2006), pre‐B, and newly generated, naive B cells (Johnson et al. 2002; Riley et al. 2005) is reduced with age. In contrast, myelopoiesis is sustained with age (Pioli et al. 2019). The loss of B cell progenitors over time is due in part to changes in the marrow environment, because lymphoid biased hematopoietic stem cells from old mice have normal B cell potential following transplantation into young animals (Montecino‐Rodriguez et al. 2019). A better understanding of why B cell development declines with age would provide new insights into the identity of these environmental signals and how aging affects them. This information would also be relevant to situations, such as following marrow transplantation, where rejuvenation of B cell development is needed.

An additional view is that the age‐related loss of B cell progenitors is due to the accumulation of inhibitory signals, such as increased inflammation (Kennedy and Knight 2017; Pietras 2017; Pioli et al. 2019), in the environment. This conclusion is supported by reports that inflammatory cytokines that include tumor necrosis factor (Ratliff et al. 2013), interleukin‐1 (Dorshkind 1988), and colony stimulating factors (Dorshkind 1991) can directly or indirectly inhibit B lymphopoiesis in vitro or in vivo. The increased production of these cytokines with age can be promoted by sustained Toll Like Receptor 4 (TLR4) signaling (Kim et al. 2023). This occurs because of increased gut permeability, which results in increased levels of circulating TLR4 ligands such as lipopolysaccharide (LPS). Thus, chronic TLR4 signaling with age is thought to be a major contributor to increased inflammation (Kovtonyuk et al. 2022), which has been termed inflammaging (Fulop et al. 2023).

If inflammaging drives the loss of B cell progenitors, then reducing inflammatory cytokine levels should attenuate this decline. However, we previously reported that aggressive treatment of old C57BL/6 (B6) mice with IL‐1 and TNF‐α inhibitors failed to restore B lymphopoiesis even incrementally (Pioli et al. 2019). This result indicated that the contribution of inflammaging to the age‐related decline of B cell development needed to be re‐evaluated and suggested that other changes in the marrow environment instead of, or in addition to, inflammation contributed to the loss of B cell progenitors over time.

There is an extensive literature indicating that B lymphopoiesis is dependent on signals from osteo‐lineage cells (Panaroni and Wu 2013; Shen et al. 2021; Zhu et al. 2007). These bone‐derived signals are likely obligate because genetic deletions resulting in bone loss trigger attenuated B lymphopoiesis (Ding et al. 2022; Zhang et al. 2023). It is interesting in this regard that CLPs have been reported to be located near the endosteum (Ding and Morrison 2013). However, whether this is also the case for their downstream progeny is unknown because the precise distribution of B cell progenitors in the marrow has not been fully mapped, particularly in long bones. In addition, an understanding of how aging affects the distribution of B lineage cells in the marrow is also limited.

We addressed these gaps in knowledge by optimizing a tissue clearing and imaging protocol that allowed us to visualize the localization of B lineage cells in the marrow across the lifespan. We initially focused on B6 mice because B lymphopoiesis declines with age in this strain (Miller and Allman 2003; Min et al. 2006) and mice on a B6 background exhibit significant senile osteoporosis (Halloran et al. 2002). The imaging of young B6 femurs showed that most B lineage cells were in fact located near bone, and particularly trabecular bone, thus providing visual evidence in support of the studies concluding that bone supports B lymphopoiesis (Panaroni and Wu 2013; Zhu et al. 2007). The imaging of femurs from old B6 mice further showed that B lineage cells were lost from areas of the marrow in which bone had degenerated and were primarily retained only in areas where bone was still intact.

We then extended our analysis to additional mouse models that either maintained or exhibited bone loss with age. As these strains on a B6 and C3H background were also TLR4 deficient or replete, this also allowed us to measure the contribution of TLR4‐induced inflammation to B lineage aging when bone mass was maintained or lost. The results indicated that the loss or maintenance of bone is a prime determinant of whether B lymphopoiesis declined or is sustained with age. The data further showed that TLR4 signaling can inhibit B lymphopoiesis, but its effects are more subtle and primarily affect cells at the pre‐B cell stage of development. We also found that any inflammation not induced through TLR4 signaling is insufficient to block B cell development.

Taken together, the results of this study indicated that the age‐related decline of B lymphopoiesis is not due simply to the build‐up of inhibitory signals in the marrow environment but to the loss of salutary ones as well, thus providing a new perspective on how the balance between positive and negative signals regulates the level of B lymphopoiesis over the life‐span.

## 2 Results

### 2.1 B Lineage Cells Are Associated With Bone and Vasculature in the Epiphyses/Metaphases

**FIGURE 1:**
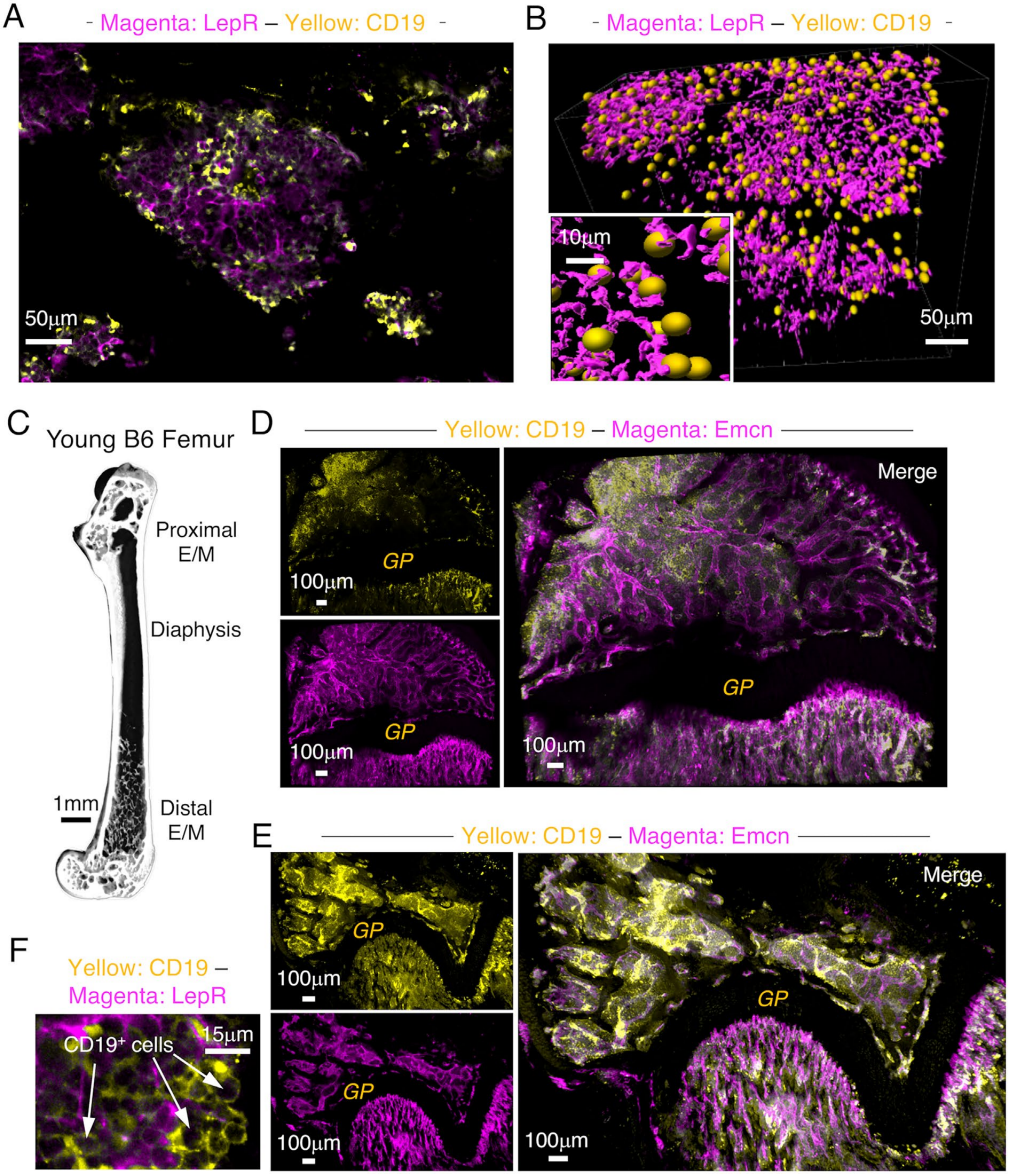
B lineage cells are associated with bone in the epiphyses/metaphases. (A) Cleared distal epiphysis from a young B6 mouse labeled with CD19 and LepR antibodies and imaged at 20×. (B) The image in panel (A) was processed with the Imaris surfaces function to visualize the three‐dimensional network of LepR^+^ stromal cells and associated CD19^+^ cells. Images shown represent results from clearing, labeling, and imaging 3–4 femurs from 3 to 4 mice. (C) Representative μCT scan of the femur from young B6 mouse. (D) Cleared proximal femur from a young B6 mouse imaged at 10× showing Emcn labelling only, CD19 labelling only, and the merged image. (E) Cleared distal femur from a young B6 mouse imaged at 10× showing Emcn labelling only, CD19 labelling only, and the merged image. (F) Digital zoom image of a single slice of an area of the tissue in panel (A). Scale bar sizes are indicated for each image. Young mice were 2–3 months old.

We combined a tissue clearing process (Greenbaum et al. 2017; Isringhausen et al. 2021; Peredo et al. 2022) that removed light scattering lipids and enabled macromolecules such as antibodies to penetrate deep into tissues (Figure S1A) with volumetric imaging to visualize the distribution of B lineage cells in B6 bone marrow. Cleared bones were labeled with antibodies to CD19, which is expressed from the pro‐B through the mature B cell stage (Hardy et al. 2007). However, surface IgM^+^ B cells account for no more than 10% of B lineage cells in the marrow of young mice, so the majority of CD19^+^ cells in that tissue are B cell progenitors (Figure S1B). CD19^+^ cells were detected by incubating cleared bones with rat anti‐mouse CD19 primary and donkey anti‐rat secondary antibodies. Bone marrow samples incubated with the secondary antibody only did not exhibit any discernible signal (Figure S1C). Adult B cell development is blocked in PU.1 hypomorphic mice (Montecino‐Rodriguez et al. 2024, 2016; Rosenbauer et al. 2006), and few CD19^+^ cells in their bone marrow were labeled with the CD19 primary and secondary antibodies (Figure S1D). We also observed few CD19^+^ cells in the lung of B6 mice. However, labeling with endomucin (Emcn) antibodies revealed the extensive vascular network in that tissue (Figure S1E). Emcn^+^ vessels frequently co‐express CD31 (Kusumbe et al. 2014), which we also observed (Figure S2A,B). We also used various computational tools on the Imaris platform to expand the information that could be obtained from two‐dimensional images. For example, use of the surfaces function revealed the intimate association between CD19^+^ B lineage and leptin receptor (LepR)^+^ stromal cells (Figure 1A,B).

The epiphyses and metaphases contain an extensive network of trabecular bone (Halloran et al. 2002), which we confirmed by micro computerized tomography (μCT) (Figure 1C), and the imaging of cleared B6 femurs labeled with CD19 antibodies revealed that numerous, dense clusters of CD19^+^ cells were abundant in these regions (Figure 1D,E). The low magnification imaging did not allow the resolution of individual CD19^+^ cells, but higher power views showed that the CD19^+^ clusters contained cells with a 6–9 μm diameter consistent with small to medium‐sized lymphocytes (Figure 1F).

Bones were also labeled with Emcn antibodies, which revealed that in addition to trabecular bone, an extensive vascular network was present in the epiphyses/metaphases (Figure 1D,E). Surface renderings of these images (Figure S2C,D) suggested that Type H blood vessels, which are branches of the arterioles that serve the bone marrow, were identified in the metaphases because of their distinctive parallel arrangement perpendicular to the growth plate (Kusumbe et al. 2014; Ramasamy et al. 2016; Sivaraj and Adams 2016). These results are in accordance with reports that the formation and maintenance of trabecular bone are dependent on a rich nutrient and oxygen supply (Kusumbe et al. 2014; Lafage‐Proust et al. 2015; Ramasamy et al. 2016).

**FIGURE 2:**
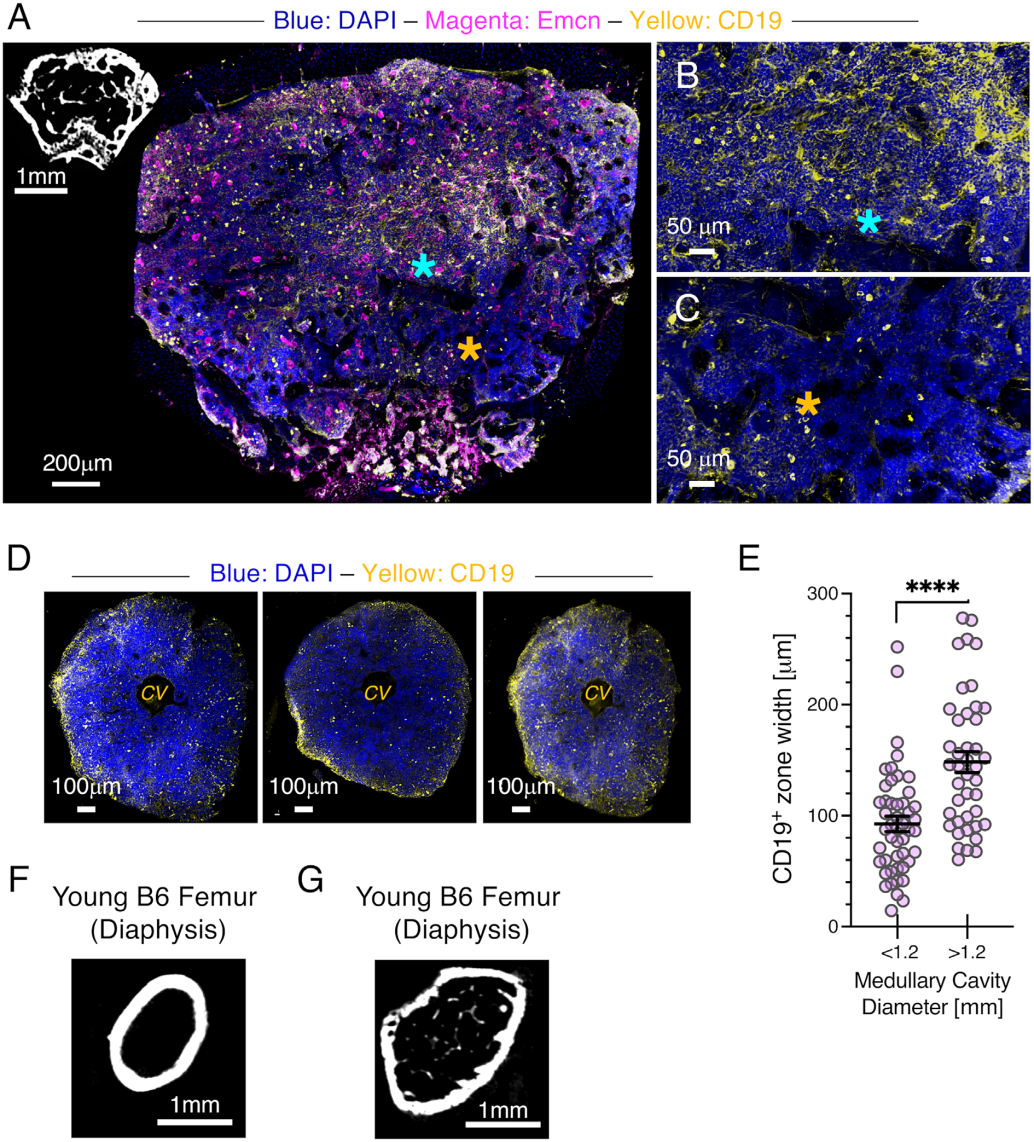
B lineage cells in the diaphysis are near the endosteal surface. (A) Cleared distal epiphysis from a young B6 mouse labeled with DAPI and antibodies to Emcn and CD19 and imaged at 10×. The insert is a representative μCT scan showing the distal epiphysis from a young B6 mouse. Images shown represent results from clearing, labeling, and imaging 4 separate femurs from 4 mice. (B) Digital zoom image of the indicated region (blue asterisk, panel A) showing a region rich in CD19^+^ cells. (C) Digital zoom image of the indicated region (orange asterisk, panel A) showing a region relatively devoid of CD19^+^ cells. (D) Cross sections of the femoral diaphysis labeled with DAPI and antibodies to CD19 showing that the width of the CD19^+^ zone of cells is variable. The samples were imaged at 20×. CV = central vein. The medullary cavity in these images is encircled by diaphyseal bone which appears black, so the CD19^+^ are located near the endosteum. (E) Quantification of width of CD19^+^ zone of cells in slices of the diaphysis where the medullary cavity diameter was less or greater than 1.2 mm. (F) Representative μCT section showing that little if any trabecular bone is present in the areas of the femoral diaphysis where the medullary cavity diameter is the narrowest. (G) Representative μCT scan showing trabecular bone in the areas of the femoral diaphysis where the medullary cavity diameter is greatest. Scale bar sizes are indicated for each image. Young mice were 2–3 months old.

B lineage cells were often regionalized in distinct areas in the epiphyses/metaphyses. For example, CD19^+^ cells were observed near the growth plate in many images (Figure 1D,E, Figure S2E,F), but whether this means areas of developing bone provide a salutary environment for B lymphopoiesis remains to be determined. The segregation of CD19^+^ cells in distinct domains was particularly apparent in the epiphyses. Cross‐sectional images of the distal epiphysis showed the existence of distinct DAPI^+^ CD19^+^ and DAPI^+^ CD19^−^ clusters and further highlighted their preferential distribution (Figure 2A–C). Stromal cells with bone‐forming potential provide a niche for CLPs (Ding and Morrison 2013; Shen et al. 2021), and these osteoprogenitors tend to be positioned near arterioles and Type H vessels (Kusumbe et al. 2014). Whether the localization of these supporting stromal cells in distinct areas of the epiphysis contributes to the regionalization of CD19^+^ cells remains to be determined.

### 2.2 CD19 ^+^ Cells in the Diaphysis Are Located Near the Endosteum

The diaphysis contains limited trabecular bone that is generally confined to the transitional zones between the metaphyses and the diaphysis proper. The midpoint of the diaphysis contains few trabeculae (Figure 1C). Diaphysis cross sections labeled with DAPI and anti‐CD19 antibodies revealed that, while CD19^+^ cells were widely distributed throughout the diaphysis, they were concentrated near the endosteum (Figure 2D). Measurements of the width of the CD19^+^ zone (Figure S1F) and diameter of the medullary cavity revealed a general, although not absolute, correlation between these parameters. In this regard, the narrowest zones of CD19^+^ cells were most frequently observed in the narrowest portion of the diaphysis, while the widest zones were found in the areas of the diaphysis with a diameter > 1.2 mm (Figure 2E). μCT images showed that the narrowest regions of the diaphysis contained little, if any, trabecular bone (Figure 2F), while the higher diameter regions were generally metaphysis adjacent and contained trabecular bone (Figure 2G).

### 2.3 The Number of B Cell Progenitors in Each Epiphysis/Metaphasis Is the Same as in the Diaphysis

**FIGURE 3:**
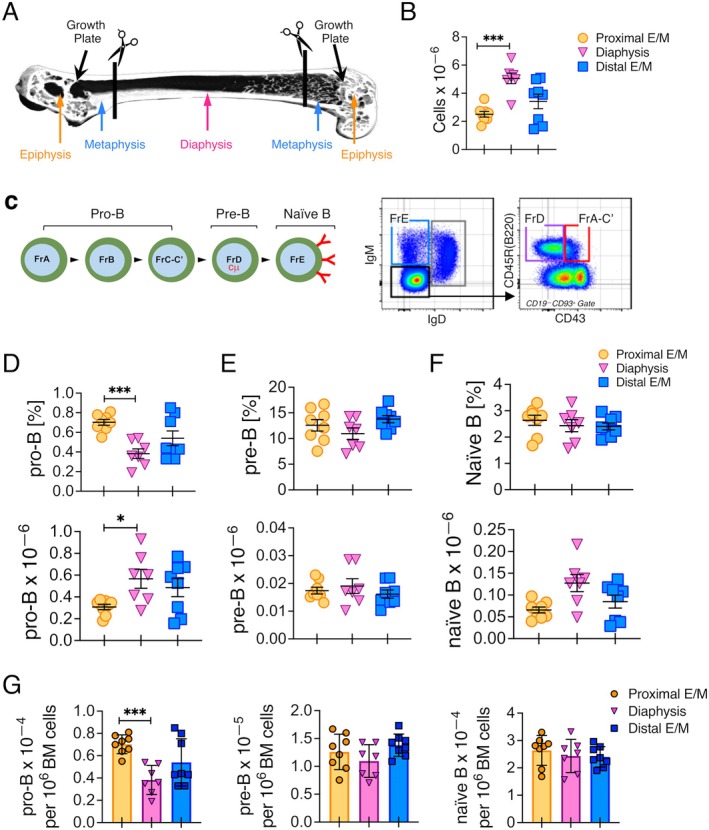
B cell progenitors are abundant in the epiphyses/metaphases. (A) Representative μCT scan of the femur from a young B6 mouse showing where cuts were made to obtain the proximal and distal epiphysis/metaphysis and the diaphysis. The cuts were made to isolate 1.5–2 mm regions of the proximal epiphysis/diaphysis and the distal epiphysis/diaphysis. The diaphysis length was ~10 mm. (B) Number of cells harvested from the indicated regions of the femur. (C) Scheme of B cell development and the gating strategy used to resolve pro‐B (Fractions A–C'), pre‐B (Fraction D), and naive B (Fraction E) cells. Frequency and number of (D) pro‐B, (E) pre‐B, and (F) naive B cells in the indicated regions of the femur. (G) Number of pro‐B, pre‐B, and naive B cells normalized to 10^6^ cells in the indicated regions of the femur. Each symbol represents an individual mouse. The young (2–3 months old) mice analyzed included 5 females and 3 males. *p < 0.05; ***p < 0.001.

We next harvested hematopoietic cells from the femur and used flow cytometry to confirm that most of the B lineage cells present in the various regions were progenitors and not mature B cells. Femurs from 13‐week‐old B6 mice were cut into three sections (Figure 3A) and total cellularity (Figure 3B) as well as the number of B cell progenitors in the epiphyses/metaphases and diaphysis were enumerated as described (Montecino‐Rodriguez et al. 2016; Pioli et al. 2019) based on the phenotypes defined by Hardy and associates (Hardy et al. 2007) (Figure 3C).

All regions contained pro‐B, pre‐B, and naive B cells. The previously described difference in the number of B cell progenitors that occurs between syngeneic B6 mice was also observed in our analyses. This variation is unlikely to be sex‐related because it is evident in both male and female animals regardless of age (Miller and Allman 2003). Because male and female mice exhibited a similar pattern of hematopoietic system aging, they were used interchangeably in all of our studies. Our analyses showed that on average the frequency and number of B lineage cells in each epiphysis/metaphysis was similar to that in the diaphysis (Figure 3D–F). This was striking because the diaphysis constituted more than 60% of the length of the femur. To account for the difference in cell recovery from each region (Figure 3B), we normalized the number of each progenitor stage per million cells in each region, and the data confirmed that pro‐B, pre‐B, and naive B cells were present at the same level in the epiphyses/metaphases as in the diaphysis (Figure 3G). In fact, the frequency and number of pro‐B cells per million marrow cells was significantly higher in the proximal epiphysis/metaphysis compared to the diaphysis, but whether this is due to a higher density of trabecular bone in the former region remains to be determined. Taken together, these data support the conclusion that developing B lineage cells are present in areas rich in trabecular bone. The results further indicated that cutting off and discarding the ends of long bones to facilitate flushing of hematopoietic cells discards most B cell progenitors.

### 2.4 B Lineage Cells Are Depleted From Areas in Which Bone Is Lost During Aging

By middle age, B6 mice exhibit a block at the pro‐B to pre‐B cell transition, resulting in an increased frequency of pro‐B cells and a loss of pre‐B cells (Miller and Allman 2003; Min et al. 2006). However, with increasing age, the frequency of pro‐B cells is lower in old compared to young mice (Miller and Allman 2003). We quantified B cell progenitors in the proximal and distal epiphyses/metaphases. While the loss of pro‐B and naive B cells in the different regions varied and was not always significantly different between young and old mice, a consistent finding was that the frequency of pre‐B cells was significantly lower in all regions of the femur from old compared to young B6 mice (Figure S3A).

**FIGURE 4:**
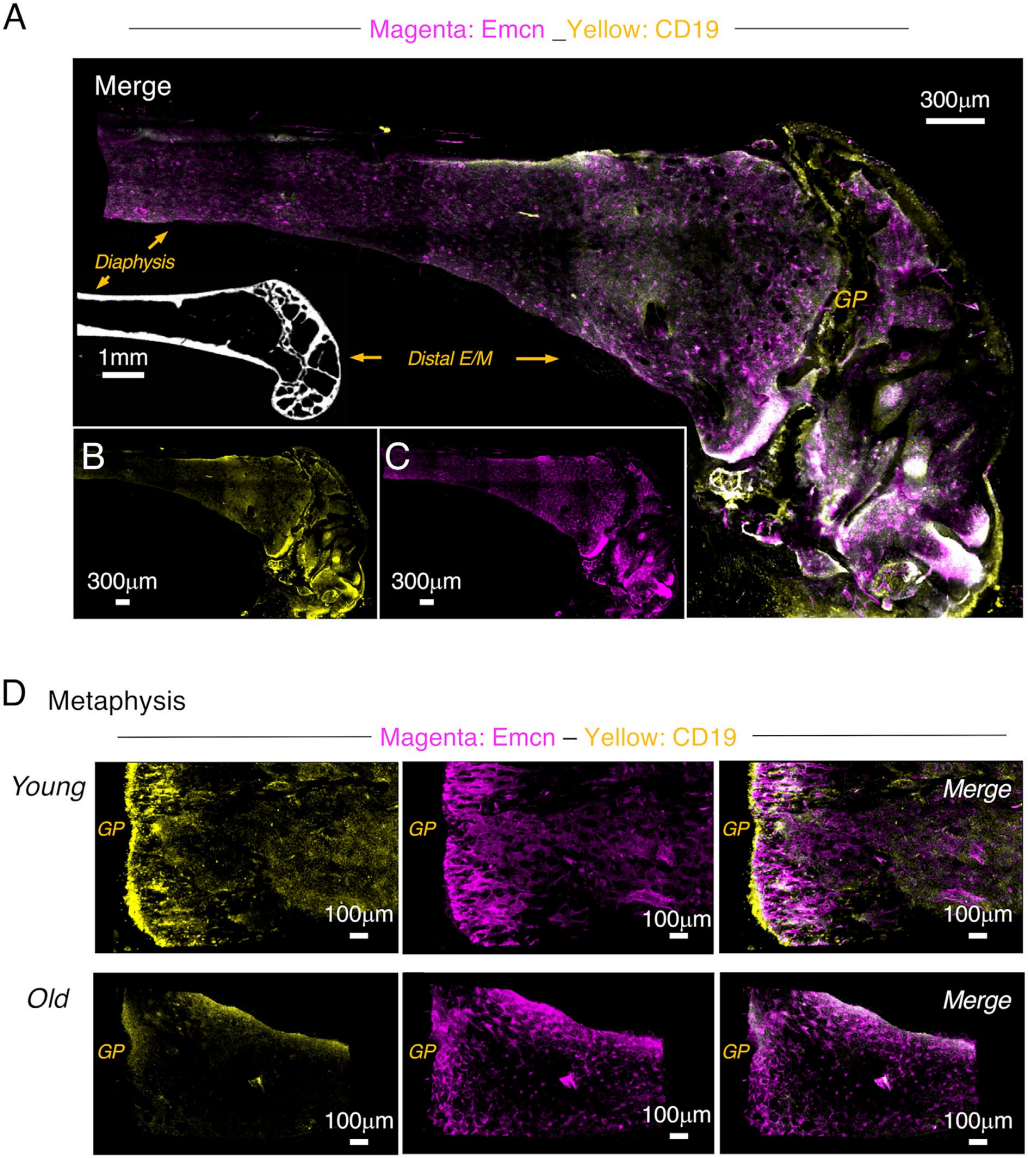
CD19^+^ cells are lost from regions where bone and vasculature deteriorate. (A) Cleared distal metaphysis from an old B6 mouse showing merged labeling with Emcn and CD19 antibodies and imaged at 10×. The insert is a representative μCT scan showing the distal region of the femur from an old B6 mouse. Images showing (B) CD19 labeling only and (C) Emcn labeling only of the merged image shown in (A). Images shown in the panels are representative from clearing, labeling, and imaging 6 separate femurs from 6 mice. (D) Cleared proximal metaphysis from young and old mice labeled with Emcn and CD19 antibodies. The Emcn labeling only, CD19 labeling only, and the merged image are shown. Images in panel D represent results from clearing, labeling, and imaging 4 and 6 separate femurs from 4 young and 6 old mice, respectively. Scale bar sizes are indicated for each image. Young mice were 2–3 months old. Old mice were 16–18 months old.

We imaged large regions of the distal metaphysis from old B6 mice (Figure 4A–C) to visualize the localization of the remaining B lineage cells. In contrast to what was observed in young mice where CD19^+^ cells were present several hundred μm from the growth plate towards the diaphysis (Figures 1D,E and 4D, Figure S2D), CD19^+^ cells in the old metaphysis were primarily located near the endosteum (Figure 4A,B,D). μCT scans further revealed that the scarcity of CD19^+^ cells in the central regions of the metaphysis correlated with a loss of trabecular bone in that region (Figure 4A, insert; Figure S3B), consistent with previous reports that B6 mice develop severe senile osteoporosis (Glatt et al. 2007; Halloran et al. 2002; Xu et al. 2021). In contrast to the severe loss of CD19^+^ cells in the metaphysis, they were more widely distributed in the epiphyses of old mice (Figure 4A,B), consistent with the fact that trabecular bone remained in that region of the femur (Figure 4A, Figure S3B).

The vasculature has been reported to deteriorate with age in B6 mice (Kusumbe et al. 2014, 2016). The labeling of cleared femurs from old B6 mice with antibodies to Emcn confirmed those reports. We observed that presumptive Type H vessels were disorganized in the metaphysis of old B6 mice (Figure 4D, Figure S3C), but the analysis of these images using surface rendering revealed that vessels that likely included sinusoids were present throughout the metaphysis, including in areas where bone loss had occurred (Figure S3B). However, the surface rendering image (Figure S3C) again showed that CD19^+^ cells were generally absent from these regions and localized to areas where bone remained, suggesting that vasculature alone inefficiently supported B cell development.

### 2.5 TLR4 Deletion Does Not Rescue B Cell Development in B6 Mice

**FIGURE 5:**
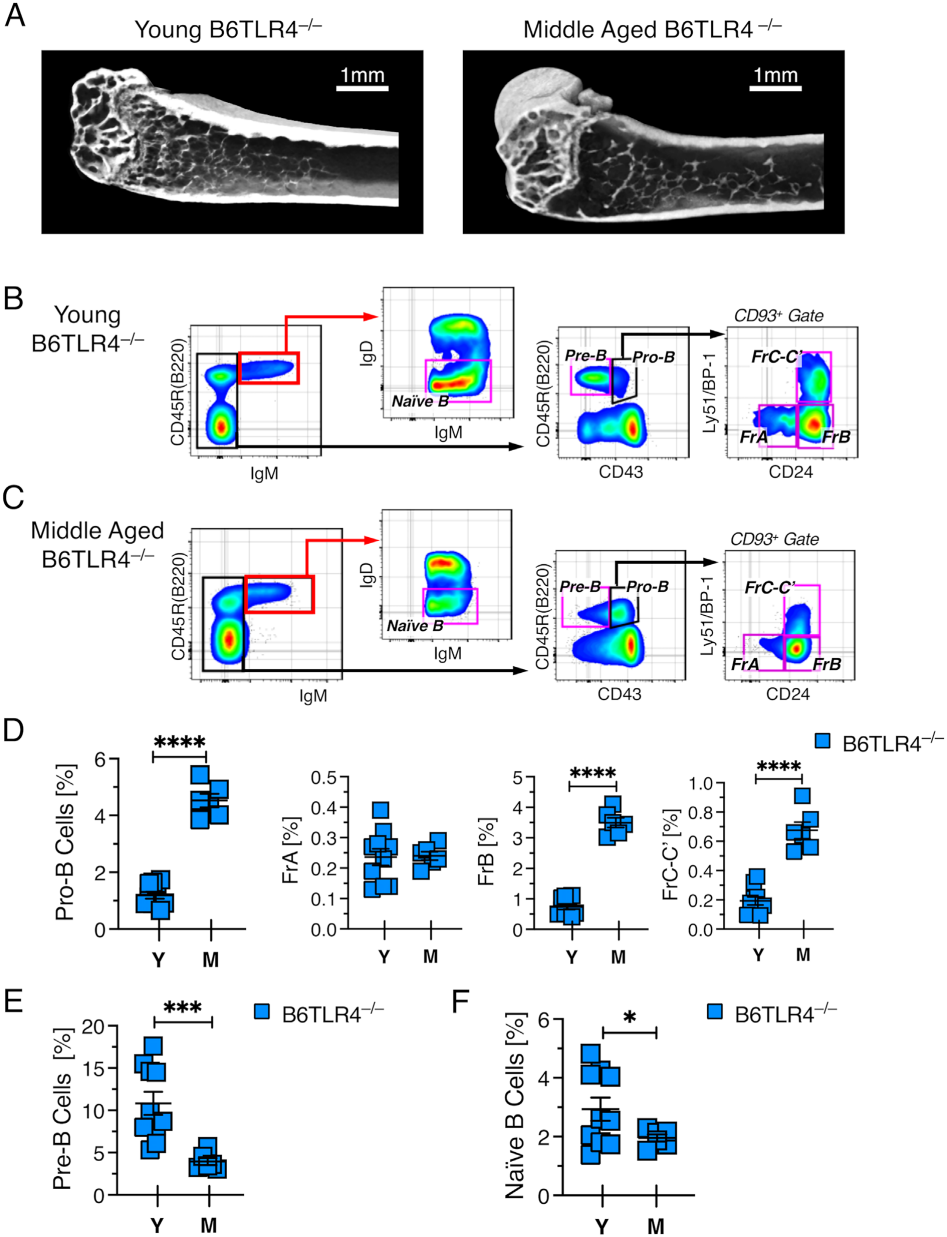
B cell development declines with age in B6TLR4^−/−^ mice. (A) Representative μCT scans of the distal femur from young and middle aged B6TLR4^−/−^ mice. Scale bar = 1 mm. FACS plots showing resolution of B lineage cells in (B) young and (C) middle‐aged B6TLR4^−/−^ mice. Frequency of (D) pro‐B, (E) pre‐B, and (F) naïve B cells in 2 and 10‐month‐old B6TLR4^−/−^ mice. The 2 month old mice were all females and the 10 month old mice were all males. Each symbol in panels D–F indicates an individual mouse. Young mice were 2–3 months old. Middle‐aged mice were 10 months old. *p ≤ 0.05; ***p ≤ 0.001; ****p ≤ 0.0001.

The above data implicated bone loss as a key driver of B lineage aging. However, as TLR4 signaling is intact in wild‐type B6 mice, TLR4‐induced signals could have been responsible for the observed loss of B cell progenitors. We assessed this by examining B cell development in B6 mice with a deletion of *Tlr4*, the gene that encodes TLR4. μCT scans of the distal femur showed that, consistent with their B6 background, trabecular bone was less abundant in B6TLR4^−/−^ mice by middle age and that spacing between the remaining trabeculae had increased (Figure 5A). Thus, B6TLR4^−/−^ mice allowed us to assess the impact of bone loss on B cell development in the absence of TLR4 signaling. Middle aged B6TLR4^−/−^ mice had a block at the pro‐B to pre‐B cell transition (Figure 5B,C) resulting, as discussed above, in a significant elevation in the number of pro‐B cells (Figure 5D) as previously reported (Miller and Allman 2003); thus, the absence of TLR4 signaling did not appear to change the kinetics with which age‐related changes in B lymphopoiesis occurred. There was also a significant reduction in pre‐B (Figure 5E) and naive B cells (Figure 5F). Again, even though syngeneic mice were compared, there was mouse‐to‐mouse variation in the frequency of B cell progenitors regardless of sex as previously described (Min et al. 2006; Miller and Allman 2003). Taken together, the analysis of B6TLR4^−/−^ mice showed that TLR4 deficiency failed to prevent the age‐related decline in B cell development when bone mass was not maintained.

### 2.6 B Cell Development Does Not Decline in Old C3H/HeJ Mice

**FIGURE 6:**
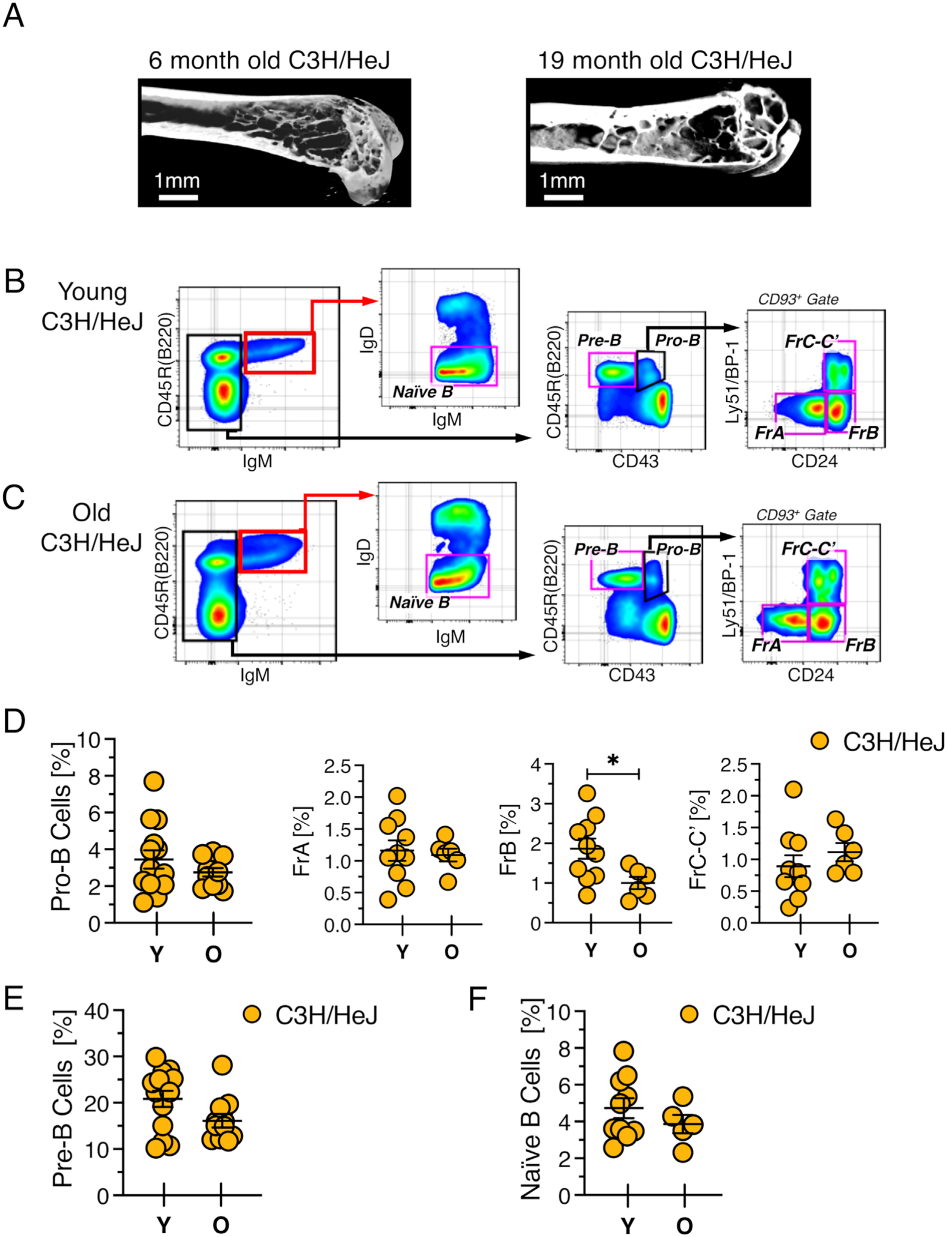
B cell development is maintained with age in C3H/HeJ mice. (A) Representative μCT scans of the distal femur from 6 and 19 month old C3H/HeJ mice. Scale bar = 1 mm. FACS plots showing resolution of B lineage cells in (B) young and (C) old C3H/HeJ mice. Frequency of (D) pro‐B, (E) pre‐B, and (F) naïve B cells from young and old female C3H/HeJ mice. Each symbol indicates an individual mouse. Young mice in panels B–F were 2–3 month and old mice were 16–18 months old. *p ≤ 0.05.

If bone loss is a major driver of B lineage aging, then the decline of B cell development should be abrogated or attenuated in strains of mice in which bone mass is maintained over time. We tested this using C3H/HeJ mice based on reports that bone volume is high in this strain (Scheller et al. 2015; Sheng et al. 1999) and it declines less precipitously with age compared to B6 mice (Scheller et al. 2015). μCT images confirmed that bone mass was extensive in old C3H/HeJ mice (Figure 6A). The comparison of B lymphopoiesis in young and old C3H/HeJ mice revealed that B cell development did not decline with age (Figure 6B,C). The frequency and number of pro‐B (Figure 6D), pre‐B (Figure 6E), and naïve B cells (Figure 6F) were similar in young and old animals. Bone marrow cellularity in C3H/HeJ mice was stable or elevated with age (Figure S4), indicating that the total number of B lineage cells also did not decline over time.

In addition to sustained high bone mass, C3H/HeJ mice are distinguished by a mutation in *Tlr4* resulting in defective TLR4 signaling (Poltorak et al. 1998). Thus, the C3H/HeJ data demonstrated that maintaining bone mass and blocking TLR4 signaling can prevent the age‐related decline of B cell development. These results further indicated that any increase in inflammation beyond levels induced by TLR4 signaling does not contribute to the age‐related loss of B cell progenitors. Otherwise, a block in B cell development should have been observed in old C3H/HeJ mice.

### 2.7 The Decline of B Lymphopoiesis Is Attenuated in Old C3H/HeOuJ Mice

**FIGURE 7:**
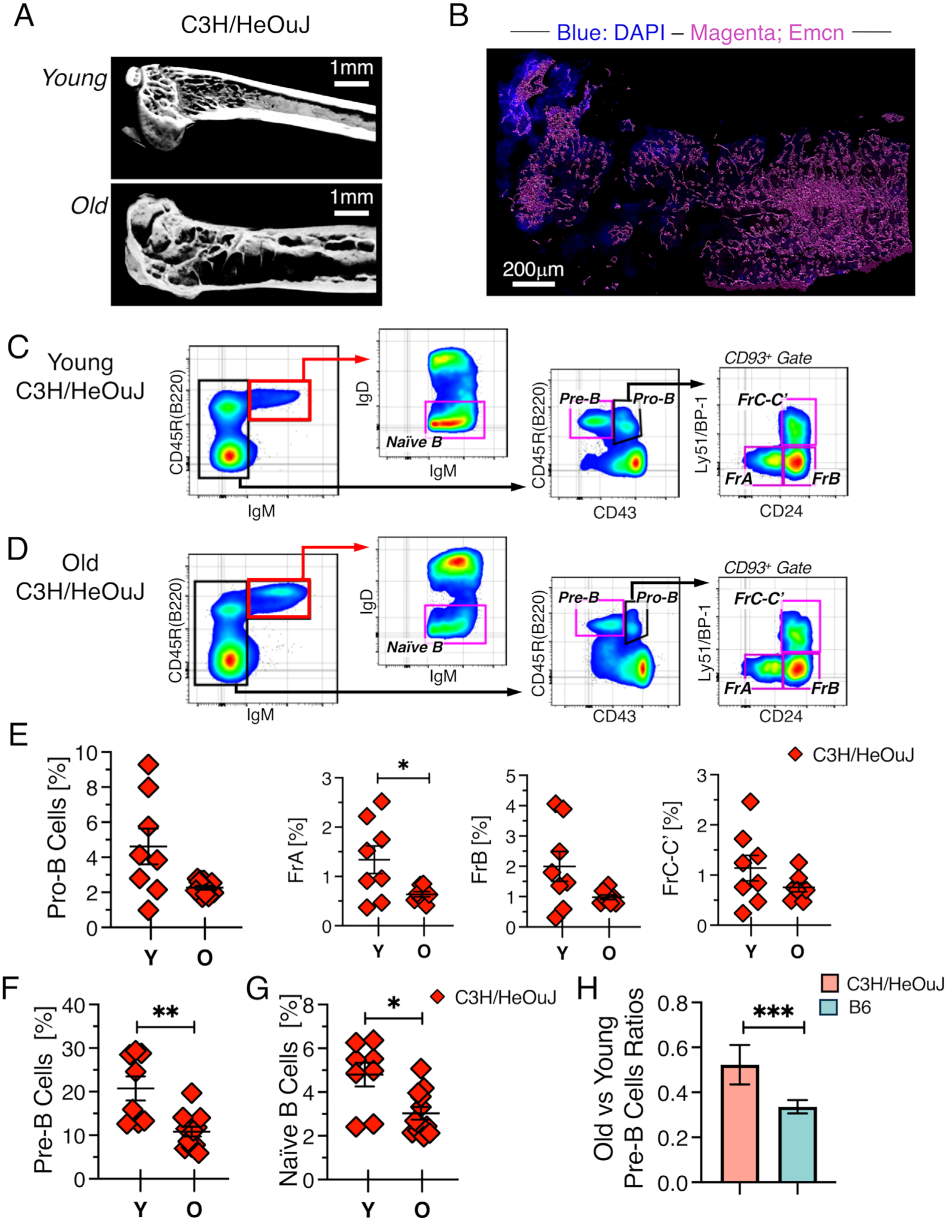
The age‐related decline in B cell development is attenuated in C3H/HeOuJ mice. (A) Representative μCT scans of femur from young and old C3H/HeOuJ mice. Scale bar = 1 mm. (B) Surface rendering showing Emcn^+^ vasculature in the metaphysis of an old C3H/HeOuJ mouse. Scale bar = 200 μm. FACS plots showing resolution of B lineage cells in (C) young and (D) old C3H/HeOuJ mice. Frequency of (E) pro‐B, (F) pre‐B, and (G) naïve B cells from young and old C3H/HeOuJ mice. (H) Comparison of old versus young pre‐B cell ratios in C3H/HeOuJ and B6 mice based on analysis of 8 mice from each strain. Each symbol indicates an individual mouse. Young mice were 2–3 month old females and old mice were 16–18 months old males. *p ≤ 0.05; **p ≤ 0.01; ***p < 0.001.

The analysis of B6TLR4^−/−^ mice indicated that abrogation of TLR4 signaling could not prevent the age‐related decline of B cell development when bone mass was not maintained. However, a remaining question was whether TLR4 signaling had any negative effect on B cell development when bone mass remained high with age. We examined TLR4 replete C3H/HeOuJ mice to address this question. C3H/HeJ and C3H/HeOuJ are genetically similar strains, and C3H/HeOuJ mice also have high femoral bone mass (Butylina et al. 2025). Our μCT scans showed that trabecular bone was maintained in the femur of C3H/HeOuJ mice with age (Figure 7A). An extensive network of blood vessels was also present in the femoral metaphysis, which was consistent with the dependence of trabecular bone formation and maintenance on vascular signals (Kusumbe et al. 2014; Lafage‐Proust et al. 2015; Ramasamy et al. 2016) (Figure 7B). Thus, C3H/HeOuJ mice allowed us to assess the impact of bone maintenance on B cell development when TLR4 signaling was intact. Quantification of B cell progenitors (Figure 7C,D) showed that the pro‐B cell compartment was largely maintained in C3H/HeOuJ mice (Figure 7E). The frequency of pre‐B (Figure 7F) and naive B cells (Figure 7G) declined significantly, but this loss was less severe compared to what occurred in mice on a B6 background (Figure 7H). These data indicated that TLR4 signals contribute to the age‐related decline of B cell development even when bone is maintained, but their effects are more stage specific than has been appreciated. Any loss of pre‐B cells due to TLR4 signaling must be indirectly mediated, because pro‐B and pre‐B cells expressed negligible levels of TLR4 (Figure S4D).

## 3 Discussion

The results of this study demonstrated that most B cell progenitors in young mice are bone adjacent, thus providing visual evidence supporting numerous reports that bone supports B cell development (Panaroni and Wu 2013; Wu et al. 2008; Zhu et al. 2007). We also found that developing B lineage cells were lost from areas of the marrow in which bone loss occurred with age and were retained only in areas in which it was maintained.

Previous imaging of small, often undefined areas of the bone marrow led to the conclusion that pro‐B and pre‐B cells are associated with stromal cells located near sinusoids; in fact, summary figures in some of these papers depict this close association between B cell progenitors and sinusoids (Cordeiro Gomes et al. 2016; Tokoyoda et al. 2004; Wu et al. 2024; Zehentmeier and Pereira 2019). Our data do not negate those results because the stromal and sinusoidal cell network in the marrow is extensive (Gomariz et al. 2018), and we also observed a few CD19^+^ cells associated with sinusoids in the more central regions of the diaphysis. However, our images clearly indicated that B lineage cells were sparse in areas of the marrow devoid of bone, indicating that perisinusoidal stromal cells alone do not provide an optimal environment for B lymphopoiesis. The Morrison laboratory reported that cells with a lineage negative, CD127 (IL‐7 Receptor)^+^ phenotype, which are likely CLPs, were located near the endosteal surface of bone (Ding and Morrison 2013; Shen et al. 2021), and our data indicate that their CD19^+^ progeny are as well. The localization of CLPs near bone has been controversial (Kondo 2016), and our data provide a new perspective as to whether they are (Ding and Morrison 2013) or are not (Cordeiro Gomes et al. 2016) located near the endosteum. In this regard, we observed that the CD19^+^ zone was quite narrow in some regions of the diaphysis, while in others it was more extensive. μCT scans of different regions of the diaphysis indicated that the narrowest zone of CD19^+^ cells was found in low diameter regions of the diaphysis that contained little trabecular bone. In contrast, the CD19^+^ zone was broader in regions of the medullary cavity that had a greater diameter and which contained trabecular bone. These observations suggest that in some areas of the diaphysis CLPs, and their downstream CD19^+^ progeny, might be close to the endosteum while in other regions they may be located at a greater distance but nevertheless close to bone. In view of these considerations, the disparate conclusions regarding the distance of CLPs from the endosteum made in previous reports (Cordeiro Gomes et al. 2016; Ding and Morrison 2013) are both likely correct depending on what area was imaged.

There are several ways in which osteolineage cells support B lymphopoiesis. For example, osteoblasts are a source of the obligate B lymphopoietic factor IL‐7 (Wu et al. 2008; Zhu et al. 2007), although not all groups agree with this finding (Cordeiro Gomes et al. 2016). Osterix expressing bone cell precursors can promote the pro‐B to pre‐B cell transition through secretion of Insulin Like Growth Factor‐I (Yu et al. 2016). They could also independently promote B lymphopoiesis through maintenance of stromal cells that support lymphocyte development (Sato et al. 2013). These observations provide a basis for understanding how loss of bone and bone cell precursors with age results in a decline in B cell development. The loss of the supportive milieu provided by osteolineage signals, along with heightened levels of inflammation, could also contribute to intrinsic defects in the few B cell progenitors present in old marrow. These include increased expression of p16^Ink4a^ and Arf that is responsible for their reduced growth and survival (Signer et al. 2008). Old B cell progenitors also have altered expression of the E2A transcription factor and surrogate light chains (Riley et al. 2005). However, these observations were based on analysis of B cell progenitors in strains that undergo bone loss with age. It would be interesting to determine if these cell intrinsic changes were abrogated in pro‐B and pre‐B cells in mice that do not undergo senile osteoporosis and have low levels of inflammaging.

In addition to bone, the areas in which CD19^+^ cells localized also contained an extensive vascular network. This was particularly evident in the epiphyses/metaphyses. The vessels oriented perpendicular to the growth plate in the metaphysis have been termed Type H blood vessels because of their high expression of CD31 and endomucin (Kusumbe et al. 2014). We did not measure the intensity of labeling with antibodies to both determinants, but our imaging revealed the presence of vessels oriented parallel to each other and perpendicular to the growth plate as described (Kusumbe et al. 2014). It is thus likely that Type H vessels were revealed in our images of young B6 mice as well. The presence of bone and Type H vessels in the same region reflects the interdependence of these two structures. For example, the high nutrients in Type H vessels, which are direct branches of the arteries that serve the bone marrow (Chen et al. 2020; Filipowska et al. 2017; Prisby 2017), support the formation and maintenance of trabecular bone (Kusumbe et al. 2014; Lafage‐Proust et al. 2015; Peng et al. 2020; Ramasamy et al. 2016). Osteoblasts in turn regulate angiogenesis and Type H vessels in a paracrine fashion (Tuckermann and Adams 2021). Thus, loss of Type H vessels leads to bone loss and bone loss leads to loss of Type H vessels. Regardless of which occurs first, a vicious cycle is initiated that results in a loss of trabecular bone that is deleterious to B cell development. Since Type H vessels are nutrient‐rich and B cell progenitors are metabolically active (Akkaya and Pierce 2019; Stein et al. 2017; Zhang et al. 2022), their deterioration with age (Kusumbe et al. 2014; Kusumbe et al. 2016) may further compromise B cell development. However, vessels remained throughout the metaphases in old animals, but CD19^+^ cells were nevertheless absent, suggesting that the vasculature in the absence of bone inefficiently supported B cell development.

We also analyzed how the balance between bone and TLR4 signaling contributed to the age‐related decline in B lymphopoiesis. The examination of distinct strains on a B6 and C3H background allowed us to quantify B cell progenitors in an environment in which bone mass was high or low and TLR4 expression was present or absent with age. The results obtained with C3H/HeJ mice demonstrated that the age‐related loss of B cell progenitors could be totally prevented when bone mass was maintained and TLR4 signaling was absent. One additional conclusion from the C3H/HeJ data is that increases in inflammation not dependent on TLR4 signaling are insufficient to inhibit B cell development. If this had been the case, B cell development should have been depressed in C3H/HeJ mice.

However, when bone mass declined or TLR4 signaling was intact, a loss of B cell progenitors occurred. For example, even though TLR4 signaling was blocked in B6TLR4^−/−^ mice, they had bone loss and a significant decline in the number of B cell progenitors by middle age. Similarly, a loss of B cell progenitors occurred in C3H/HeOuJ mice because, even though bone mass was high across the life span, TLR4 signaling was intact. Nevertheless, the C3H/HeOuJ data indicated that the effects of TLR4‐induced signals on B cell progenitors are nuanced. In this regard, the pro‐B cell compartment was largely intact in old TLR4‐replete C3H/HeOuJ mice, indicating that TLR4 signaling had only modest effects on B cell progenitors at this stage of development. Thus, bone‐derived signals, some of which could be provided through direct contact between B cell progenitors and osteolineage cells (Zhu et al. 2007), are sufficient to maintain the pro‐B cell compartment even when TLR4 signaling occurs. In contrast, there was a significant loss of pre‐B cells in old C3H/HeOuJ mice, indicating that TLR4 signals can impact this stage of development even when bone mass is maintained. This could be due to a block of the pro‐B to pre‐B cell transition and/or effects on the survival of newly produced pre‐B cells. However, it is important to emphasize that the magnitude of the pre‐B cell loss in C3H/HeOuJ mice was not as severe as that which occurred in wild‐type B6 mice.

Because TLR4 signals can contribute to inflammaging (Kovtonyuk et al. 2022) and inflammatory cytokines can inhibit B cell development (Pietras 2017), a conservative conclusion is that the *Tlr4* defect in C3H/HeJ mice contributed to sustained B lymphopoiesis in that strain while TLR4 signals drove the loss of pre‐B cells in C3H/HeOuJ mice. Our data showing that pro‐B and pre‐B cells did not express TLR4 indicates that binding of TLR ligands to non‐B lineage cells in the marrow and/or systemic environment impacted the pre‐B cell compartment indirectly. However, whether genetic differences in addition to *Tlr4* expression underlie our results arises because C3H/HeJ and C3H/HeOuJ mice are not syngeneic. We consider this unlikely because very few genetic differences in addition to *Tlr4* exist between the two strains. C3H/HeJ but not C3H/HeOuJ mice express the Spike Wave Discharge 1 gene (Tokuda et al. 2009). They also have an inversion on chromosome 6 that is not associated with any phenotype (Akeson et al. 2006). Finally, the transposable repetitive element (TREome) landscape also differs between these strains, but this is also the case with syngeneic wild type and C57BL/6 knockout mice (Lee et al. 2016). However, there is no evidence that any of these differences affect B cell development.

Our data suggested that the effects of inflammaging on the decline of B cell development are limited. This conclusion seems at odds with a general view that inflammation blocks B cell development (Pietras 2017) and that cytokines such as interleukin‐1 (Dorshkind 1988) and colony stimulating factors (Dorshkind 1991) broadly inhibit B lymphopoiesis in vitro and in vivo. However, a role for inflammation in blocking B lymphopoiesis is based on the effects of acute, experimentally induced inflammation such as by administration of LPS or various cytokines to mice. In contrast, the chronic inflammation associated with aging has been shown to be lower grade (Helbling et al. 2019), and we suggest that inflammaging impacts B cell development only moderately. This conclusion is consistent with our aforementioned data showing that simultaneous treatment of old B6 mice with IL‐1 and TNF‐α inhibitors failed to rejuvenate B cell development (Pioli et al. 2019). Interestingly, the latter treatments in old mice reduced the elevated myeloid progenitor levels to what was observed in young animals, indicating that inflammaging contributed to the age‐associated myeloid skewing.

We considered additional explanations for why B cell development was generally intact in old C3H compared to B6 mice. There is no reason to conclude that differences in the maintenance of the overall stromal cell network were responsible because, except for the loss of stromal cells with osteolineage potential (Shen et al. 2021), the number and organization of CXCL12 expressing stromal cells are stable over time in B6 mice (Gomariz et al. 2018). On the other hand, levels of insulin‐like growth factor‐1 (IGF‐I) are high in C3H compared to B6 mice (Iida et al. 2005), and IGF‐I can synergize with IL‐7 to increase pro‐B cell proliferation (Billips et al. 1992; Landreth et al. 1992). Thus, high levels of IGF‐I could directly support B cell development independent from other signals. However, IGF‐I also promotes bone growth and maintenance (Tahimic et al. 2013), and osterix^+^ osteoprogenitors are a source of IGF‐I in the marrow (Yu et al. 2016). Thus, when the differences between B6 and C3H background mice are considered, whether osteolineage cells are maintained with age remains the unifying element that explains differences in B cell development between these strains.

Our results demonstrate that analysis of multiple mouse strains is critical for understanding the signals that regulate B cell development across the life span. It is interesting to consider that if various inbred mouse strains were combined blindly and then sampled, some animals would show a loss of B cell progenitors with age while B cell development would not decline in others. A report that examined B cell development in young and old humans also showed that B lymphopoiesis was stable in many individuals while others exhibited a loss of lymphocyte precursors with age (Rossi et al. 2003). Thus, the combined examination of various inbred mouse strains could serve as a basis for understanding why some elderly humans exhibit a decline in B lymphopoiesis with age and others do not. In any case, the current results support a model in which loss of salutary signal(s) provided by bone is a principal driver of the age‐related decline of B lymphopoiesis. This conclusion provides a new perspective as to why the latter process deteriorates with age, because the prevailing model is that it is due to increased inhibitory signals. The results also provide a clear example of how multiplexed and multiscaled volumetric imaging can be employed to visualize B cell development and how changes in the environment compromise that process.

## 4 Methods

### 4.1 Mice

C57BL/6 (B6), B6 (Cg)‐*Tlr4* ^*tm1.2Karp*^/J, C3H/HeJ, and C3H/HeOuJ male and female mice were obtained from the Jackson Laboratory (Bar Harbor, ME) and maintained in the UCLA Division of Laboratory Animal Medicine. Some old B6 mice were obtained from the National Institute on Aging aged rodent colony. Breeding pairs of PU1 hypomorphic (Sfpi1tm1.3Dgt/J; UREΔ/Δ) mice were originally obtained from the Jackson Laboratory and were backcrossed with B6 mice for at least seven generations in the UCLA Division of Laboratory Animal Medicine. Mice were housed in the UCLA Division of Laboratory Animal Medicine vivarium, and all protocols were approved by the UCLA Institutional Animal Care and Use Committee. Young mice were 2–3 months of age. Middle‐aged mice were 10 months of age. Old mice were 16–18 months of age.

### 4.2 Harvest of Hematopoietic Cells

After dissecting away muscle and connective tissue from femurs, two cuts were made yielding 1.5–2 mm sections of the proximal and distal epiphysis/metaphysis and the diaphysis. Hematopoietic cells were isolated from each section by crushing them in Ca^++^Mg^++^ free PBS. Red blood cells were lysed using red blood cell lysis buffer for 3 min. Cell counts were performed using a hemocytometer with 1× eosin in Ca^++^Mg^++^ free PBS as a live/dead stain.

### 4.3 Flow Cytometry

All staining procedures were performed in cold Ca^++^Mg^++^ free PBS. Samples were first incubated with a CD16/32 antibody to block non‐specific binding of antibodies to cells via Fc receptors. All antibodies utilized are listed in Table S1. For surface staining, cells were incubated on ice for 30 min with the appropriate dilution of antibodies in one or two steps depending on the combinations of primary and secondary reagents used. Unbound antibodies were washed from cells with cold Ca^++^Mg^++^ free PBS. Stained samples were run on a LSRII (BD Biosciences) located in the Broad Stem Cell Research Center. Frequencies of cell populations were determined using the FlowJo Software v10. We used standard flow best practices to minimize variations between samples during acquisition: all samples were run on the same LSRII instrument with identical parameter settings to control for fluorescence intensity variation between runs.

### 4.4 μCT Imaging

Mouse bones were imaged using a high‐resolution μCT scanner (80 kVp, 130 μA, 0.017 μm voxel size, 3600 projections, 0.1 degree angular step, 500 ms exposure time) designed and built at the UCLA Crump Institute for Molecular Imaging. μCT data were reconstructed using the filter back‐projection method. Data analysis was performed with ORS Dragonfly software (version 2022.2. Comet Technologies Canada Inc., Montreal, Canada) using the Buie method (Buie et al. 2007).

### 4.5 Tissue Clearing

Our bone clearing protocol was based on the methodology described by Greenbaum et al. (2017). Following removal of all surrounding soft tissue, samples were incubated in 4% paraformaldehyde at 4^o^C for 48 h. Bones were then transferred to hydrogel monomer solution (A4P0: 4% acrylamide in PBS) for 72 h at 4°C protected from light (Yang et al. 2014). Samples were then polymerized at 40^o^C for 3 h and dissected from the surrounding hydrogel. Any remaining tendons and soft tissue were then removed by lightly sanding the bone with an engraving pen equipped with a diamond bur. Bones were then demineralized by incubation in 14% EDTA at room temperature for approximately 2 weeks with regular buffer changes. Samples were subsequently transferred to 8% SDS for delipidation at 4^o^C for approximately 8 days. Following delipidation, bones were incubated in 25% *N*,*N*,*N*′,*N*′‐tetrakis (2‐hydroxypropyl) ethylenediamine (Sigma‐Aldrich, #122262‐1L) for 48 h at 37°C to decolorize the tissue through heme group removal. Femurs were then either bisected or cut into cross‐sections prior to incubation with blocking agents (donkey serum) and primary and/or secondary antibodies. All antibodies utilized are listed in Table S1. Following antibody labeling, bones were refractive index matched in 80% glycerol for 24 h prior to imaging.

### 4.6 Imaging

Cleared and stained femurs were mounted in 80% glycerol in custom‐made 3‐dimensional chambers and equilibrated for a minimum of 4 h. Tiled Z‐stacks with 10% overlap were employed to image large areas of the bone. Samples were imaged using 10× or 20× objectives on an inverted Zeiss 880 confocal microscope in the Broad Stem Cell Research Center. Bone halves and cross‐section images were acquired at a bit depth of 16. Images were processed using IMARIS 10.0/10.1 software. Clearview deconvolution was applied to the data sets, and spots, distance, and surfaces functions were employed to highlight the relationship between cells and microarchitecture.

### 4.7 Statistical Analysis

Data are expressed as a mean ± SEM, as indicated in the figure legends. Differences between groups were tested using a two‐tailed, unpaired Student's *t*‐test (*α* = 0.05). The flow cytometry data are presented so that information about each individual mouse processed can be viewed.

## Author Contributions

Erin Baker, Encarnacion Montecino‐Rodriguez, Shili Xu, Sotirios Tetradis, Adrien Rouault, and Oscar I. Estrada performed the experiments. Erin Baker, Encarnacion Montecino‐Rodriguez, and Kenneth Dorshkind wrote and supervised the manuscript. All authors read and approved the final manuscript.

## Ethics Statement

Mice were housed in the UCLA Division of Laboratory Animal Medicine vivarium, and all protocols were approved by the UCLA Institutional Animal Care and Use Committee.

## Conflicts of Interest

The authors declare no conflicts of interest.

## Supporting information


**Figure S1:** Labeling of cleared bones with antibodies to endomucin and CD19. (A) Image of bone before and after clearing. (B) Representative flow cytometry plot showing resolution of CD19 and sIgM^+^ B cells in the bone marrow of a young B6 mouse. (C) Image of cleared femur from young B6 mouse showing no labelling with secondary donkey anti‐rat antibody only. Image shown represents results from clearing, labeling, and imaging 3 separate femurs. (D) Image showing no CD19^+^ cells were detected in cleared femur from a 24‐week‐old UREΔ/Δ mouse labeled with rat anti‐CD19 primary and donkey anti‐rat secondary antibodies. Images shown represent results from clearing, labeling, and imaging 4 separate bones. (E) Image of cleared lung from young B6 mouse labeled with CD19 and Emcn antibodies. Images shown represent results from clearing, labeling, and imaging 2 separate lungs. (F). Descriptive scheme showing how the width of the CD19^+^ zone of cells was measured using the IMARIS 10.0/10.1 software tools. Scale bar sizes are indicated for each image.
**Figure S2:** Vasculature is abundant in the epiphyses and metaphases.
(A) Surface rendering showing interconnected and separate Emcn^+^ and CD31^+^ vessels in the femur of an old B6 mouse. (B) Images with endomucin or CD31 labelling used to obtain the surface rendering in panel A. (C) Surface rendering showing Emcn^+^ vessels and CD19^+^ cells in proximal epiphysis and metaphysis of young B6 mouse. (D) Surface rendering showing Emcn^+^ vessels and CD19^+^ cells in distal epiphysis and metaphysis of young B6 mouse. (E) Cleared proximal metaphysis from a young B6 mouse labeled with Emcn and CD19 antibodies and imaged at 20X. (F) Slightly transparent surface rendering of panel (E) showing Emcn^+^ vessels and CD19^+^ domains. Scale bar sizes are indicated for each image. Young mice were 2–3 months old. Old mice were 16–18 months old.
**Figure S3:** B lineage cells are present near the endosteum in old B6 mice.
(A) Frequency of pro‐B, pre‐B, and naive B cells in the proximal epiphysis/metaphysis and the distal epiphysis/metaphasis of young female and old male B6 mice. Each symbol represents an individual mouse. **p < 0.01; ****p < 0.0001. (B) μCT image of femur from young and old B6 mouse. Scale bar = 1 mm. (C) Surface rendering showing Emcn^+^ vessels and CD19^+^ cells in the distal epiphysis and metaphysis of an old B6 mouse. Scale bar sizes are indicated for each image. Young mice were 2–3 months old. Old mice were 16–18 months old.
**Figure S4:** Bone marrow cellularity is stable or increased with age in C3H/HeJ and C3H/HeOuJ mice. Bone marrow cellularity in young and old female C3H/HeJ and young female and old male C3H/HeOuJ mice was stable and/or increased with age regardless of whether hematopoietic cells were obtained by (A) flushing or (B and C) crushing bones. The method used to obtain hematopoietic cells did not affect the frequency of B lineage cells. Hematopoietic cells were preferentially obtained using the crushing procedure, as cell yields were higher compared to flushing bones. Each symbol represents an individual mouse. **p ≤ 0.01. (D) Flow plots showing gates used to resolve pro‐B, pre‐B, immature B, and mature recirculating B cells in bone marrow. (E) Expression of TLR4 in the indicated B lineage populations. The mean ± SD population frequencies in total bone marrow are indicated in panels D and E. Five 5 month old female B6 mice were analyzed individually and a representative plot is shown.
**Table S1:** Antibodies, clone numbers used in this study and sources.

## Data Availability

The authors have nothing to report.
