# Supplementary material for: Bone Loss and TLR4 Signals Contribute Independently to B Lineage Aging
Source: Aging Cell. 2025 Oct 11;24(12):e70267. doi: 10.1111/acel.70267 (PMC12686596; doi:10.1111/acel.70267)
Supplement: Supplementary file 1 — Figure S1: Labeling of cleared bones with antibodies to endomucin and CD19. (A) Image of bone before and after clearing. (B) Representative flow cytometry plot showing resolution of CD19 and sIgM+ B cells in the bone marrow of a young B6 mouse. (C) Image of cleared femur from young B6 mouse showing no labelling with secondary donkey anti‐rat antibody only. Image shown represents results from clearing, labeling, and imaging 3 separate femurs. (D) Image showing no CD19+ cells were detected in cleared femur from a 24‐week‐old UREΔ/Δ mouse labeled with rat anti‐CD19 primary and donkey anti‐rat secondary antibodies. Images shown represent results from clearing, labeling, and imaging 4 separate bones. (E) Image of cleared lung from young B6 mouse labeled with CD19 and Emcn antibodies. Images shown represent results from clearing, labeling, and imaging 2 separate lungs. (F). Descriptive scheme showing how the width of the CD19+ zone of cells was measured using the IMARIS 10.0/10.1 software tools. Scale bar sizes are indicated for each image. Figure S2: Vasculature is abundant in the epiphyses and metaphases. (A) Surface rendering showing interconnected and separate Emcn+ and CD31+ vessels in the femur of an old B6 mouse. (B) Images with endomucin or CD31 labelling used to obtain the surface rendering in panel A. (C) Surface rendering showing Emcn+ vessels and CD19+ cells in proximal epiphysis and metaphysis of young B6 mouse. (D) Surface rendering showing Emcn+ vessels and CD19+ cells in distal epiphysis and metaphysis of young B6 mouse. (E) Cleared proximal metaphysis from a young B6 mouse labeled with Emcn and CD19 antibodies and imaged at 20X. (F) Slightly transparent surface rendering of panel (E) showing Emcn+ vessels and CD19+ domains. Scale bar sizes are indicated for each image. Young mice were 2–3 months old. Old mice were 16–18 months old. Figure S3: B lineage cells are present near the endosteum in old B6 mice. (A) Frequency of pro‐B, pre‐B, and naive B cells in [file ACEL-24-e70267-s001.docx]

**Bone Loss and TLR4 Signals Contribute Independently to B Lineage Aging**

Erin Baker^1^, Encarnacion Montecino-Rodriguez^1^, Shili Xu^2^, Sotirios Tetradis^3^, Adrien Rouault^1^, Oscar I. Estrada^1^, and Kenneth Dorshkind^1^

^1^Departments of Pathology and Laboratory Medicine and ^2^Molecular and Medical Pharmacology, David Geffen School of Medicine at UCLA and ^3^Division of Diagnostic and Surgical Sciences, UCLA School of Dentistry

Los Angeles, CA 90095

**Running Title:** Bone, TLR4, and B Lineage Aging

**Corresponding Author**

Kenneth Dorshkind

Department of Pathology and Laboratory Medicine

David Geffen School of Medicine at UCLA

10833 Le Conte Avenue

Los Angeles, CA 90095

310 206-9535

[kdorshki@mednet.ucla.edu](mailto:kdorshki@mednet.ucla.edu)

**Inventory of supporting information:**

4 Supplemental Figures and 1 Supplemental Table.

**
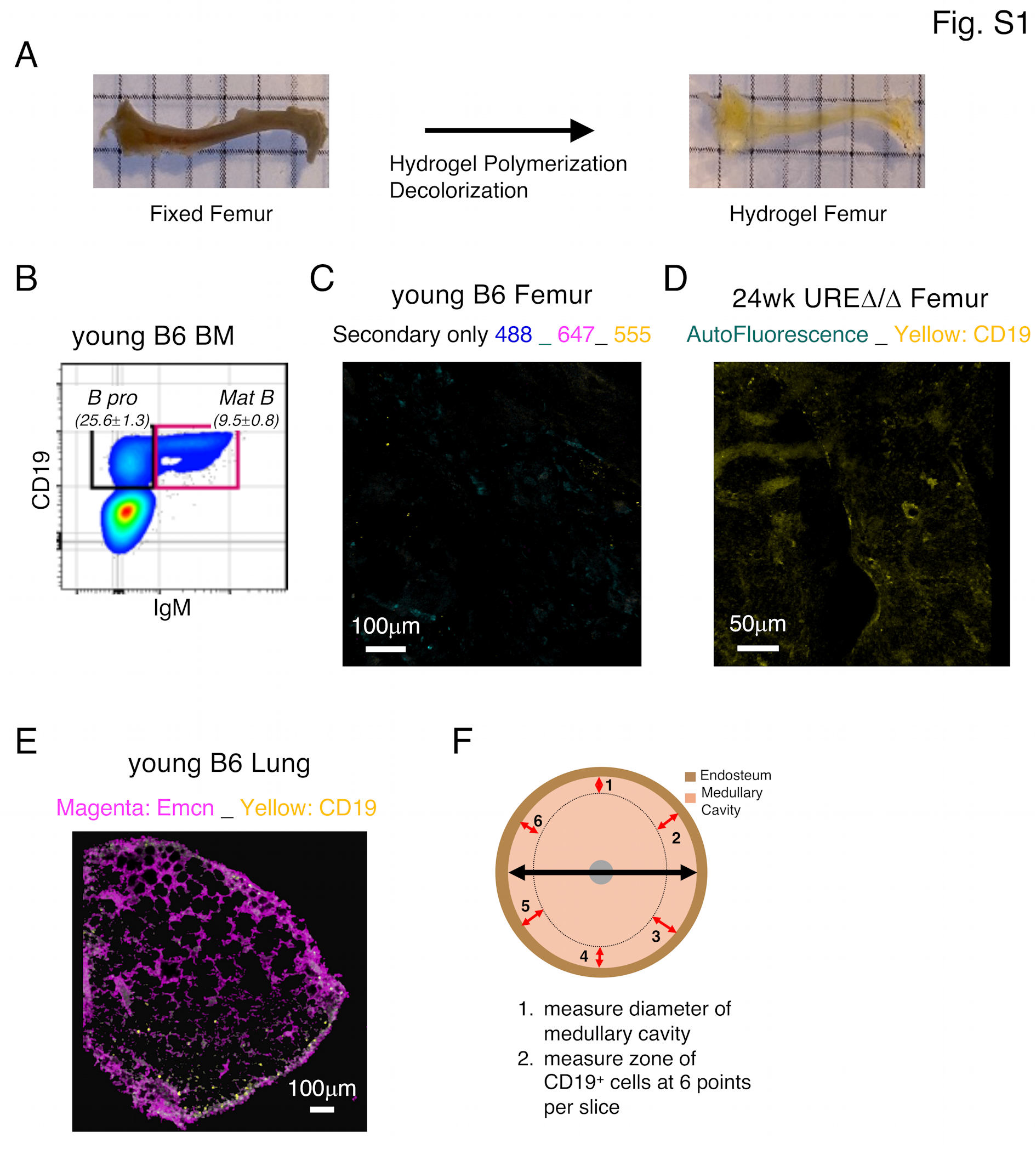
**

**Figure S1. Labeling of cleared bones with antibodies to endomucin and CD19.**

**(A)** Image of bone before and after clearing. **(B)** Representative flow cytometry plot showing resolution of CD19 and sIgM^+^ B cells in the bone marrow of a young B6 mouse. **(C)** Image of cleared femur from young B6 mouse showing no labelling with secondary donkey anti-rat antibody only. Image shown represents results from clearing, labeling, and imaging 3 separate femurs. **(D)** Image showing no CD19^+^ cells were detected in cleared femur from a 24-week-old UREΔ/Δ mouse labelled with rat anti-CD19 primary and donkey anti-rat secondary antibodies. Images shown represent results from clearing, labeling, and imaging 4 separate bones. **(E)** Image of cleared lung from young B6 mouse labeled with CD19 and Emcn antibodies. Images shown represent results from clearing, labeling, and imaging 2 separate lungs. **(F).** Descriptive scheme showing how the width of the CD19^+^ zone of cells was measured using the IMARIS 10.0/10.1 software tools. Scale bar sizes are indicated for each image.

**
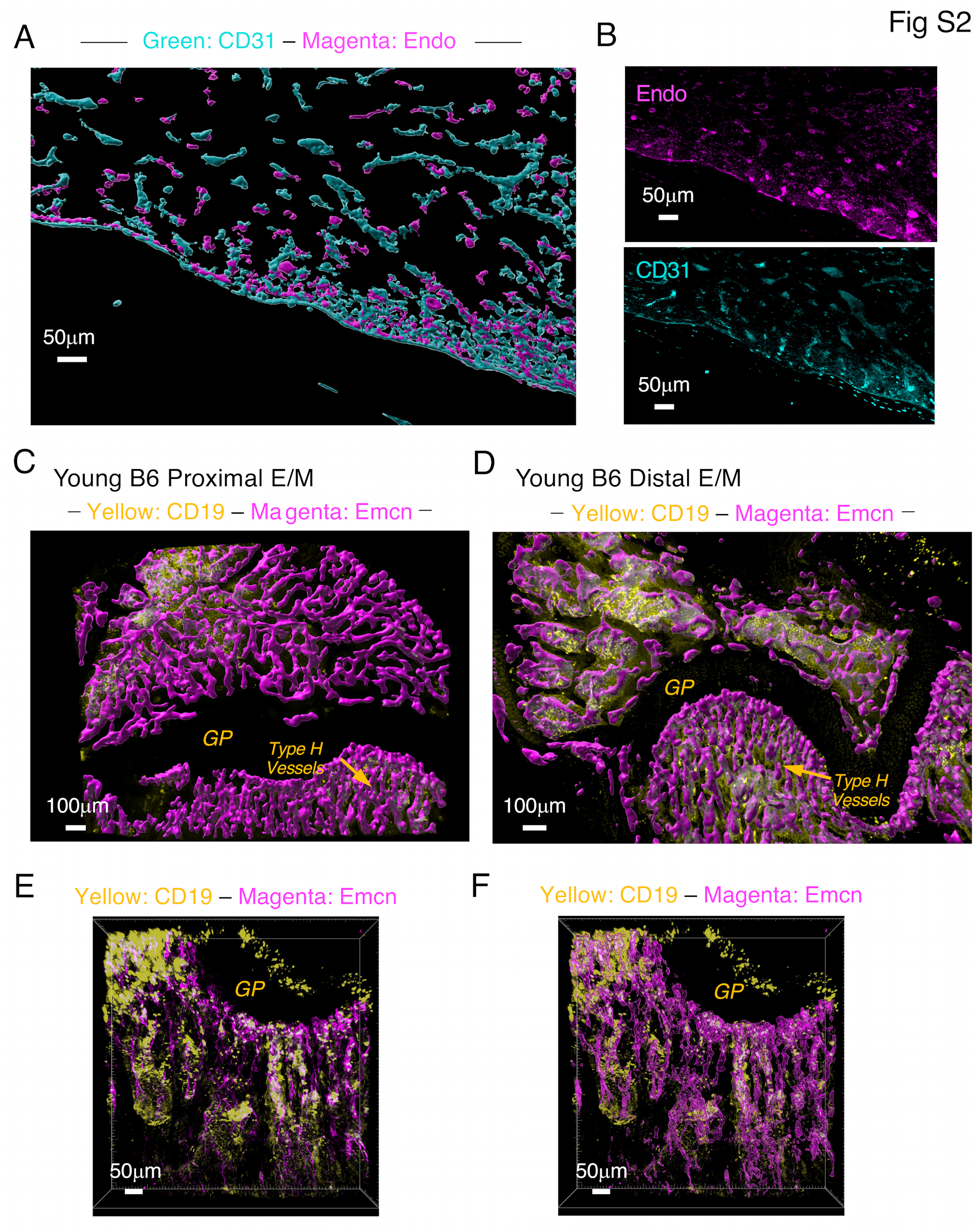
**

**Figure S2. Vasculature is abundant in the epiphyses and metaphases.**

**A)** Surface rendering showing interconnected and separate Emcn^+^ and CD31^+^ vessels in the femur of an old B6 mouse. (**B**) Images with endomucin or CD31 labelling used to obtain the surface rendering in panel A. **(C)** Surface rendering showing Emcn^+^ vessels and CD19^+^ cells in proximal epiphysis and metaphysis of young B6 mouse. **(D)** Surface rendering showing Emcn^+^ vessels and CD19^+^ cells in distal epiphysis and metaphysis of young B6 mouse. **(E)** Cleared proximal metaphysis from a young B6 mouse labelled with Emcn and CD19 antibodies and imaged at 20X. **(F)** Slightly transparent surface rendering of panel (E) showing Emcn^+^ vessels and CD19^+^ domains. Scale bar sizes are indicated for each image. Young mice were 2-3 months old. Old mice were 16-18 months old.

**
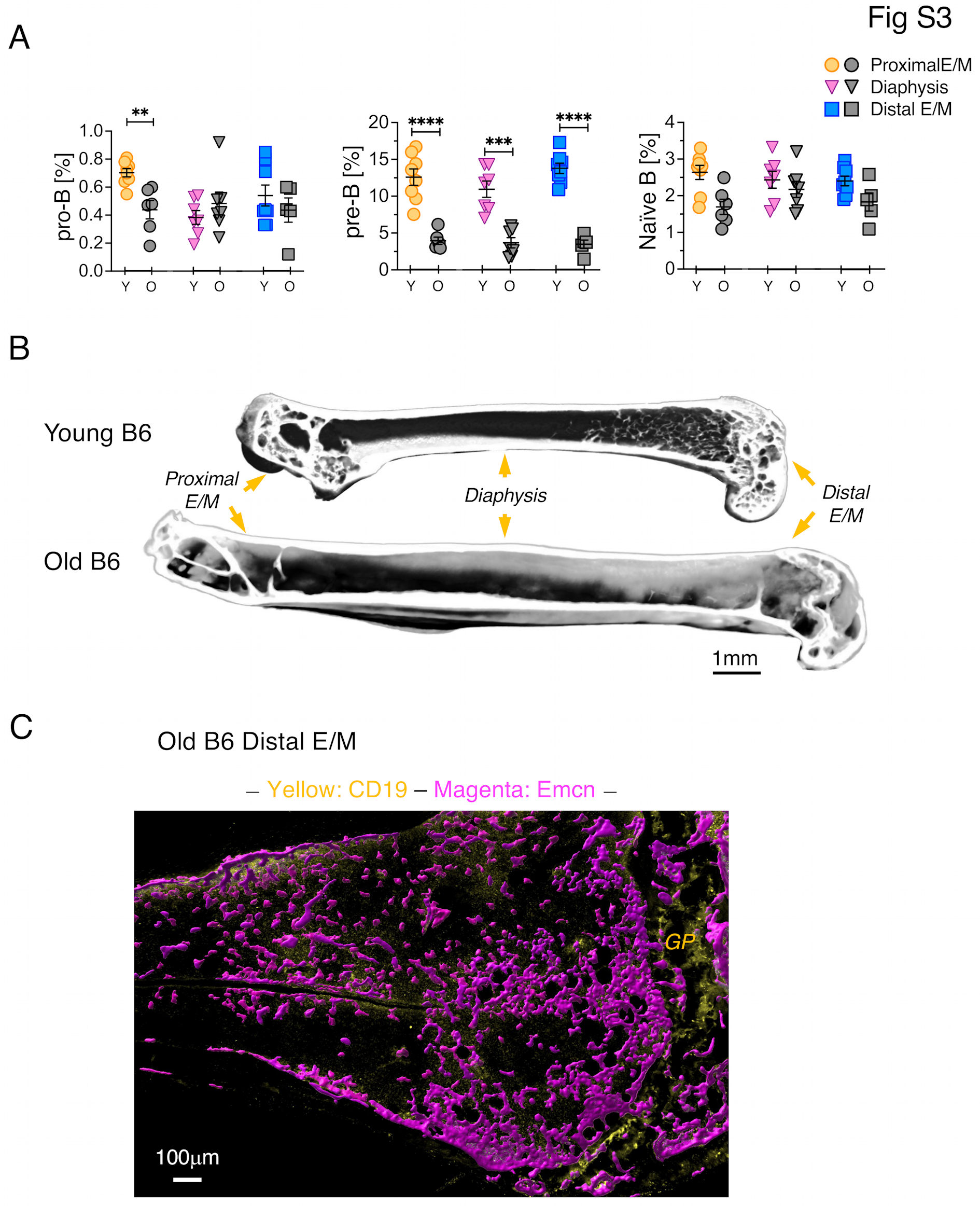
**

**Figure S3. B lineage cells are present near the endosteum in old B6 mice.**

**(A)** Frequency of pro-B, pre-B, and naive B cells in the proximal epiphysis/metaphysis and the distal epiphysis/metaphasis of young and old mice. Each symbol represents an individual mouse. **p<0.01; ****p<0.0001. **(B)** μCT image of femur from young and old B6 mouse. Scale bar = 1mm. **(C)** Surface rendering showing Emcn^+^ vessels and CD19^+^ cells in the distal epiphysis and metaphysis of an old B6 mouse. Scale bar sizes are indicated for each image.

Young mice were 2-3 months old. Old mice were 16-18 months old.

**
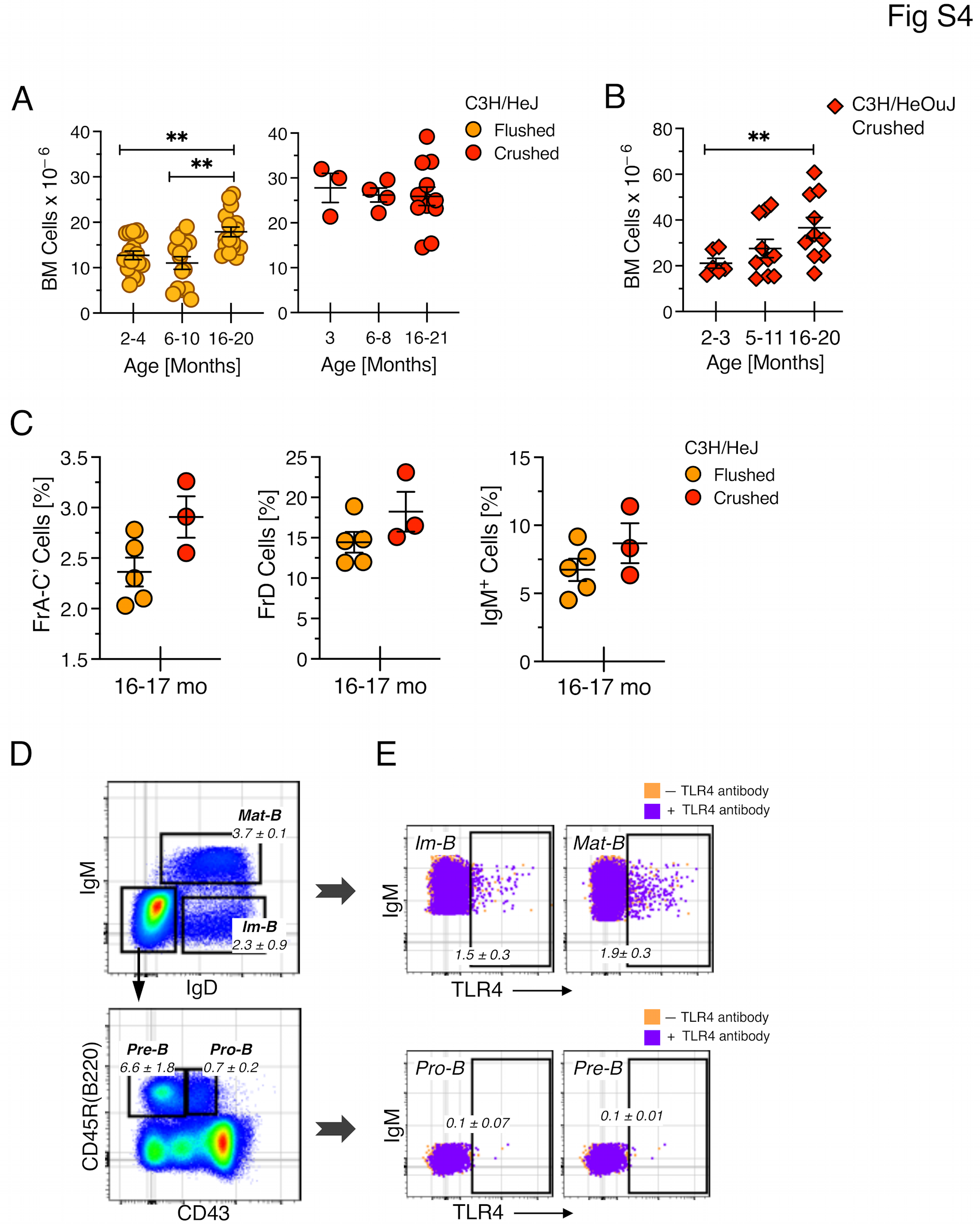
**

**Figure S4. Bone marrow cellularity is stable or increased with age in C3H/HeJ and C3H/HeOuJ mice.** Bone marrow cellularity in young and old females C3H/HeJ and young females and old males C3H/HeOuJ mice was stable and/or increased with age regardless of whether hematopoietic cells were obtained by **(A)** flushing or **(B)** and **(C)** crushing bones. The method used to obtain hematopoietic cells did not affect the frequency of B lineage cells. Hematopoietic cells were preferentially obtained using the crushing procedure, as cell yields were higher compared to flushing bones. Each symbol represents an individual mouse. **p ≤ 0.01. **(D)** Flow plots showing gates used to resolve pro-B, pre-B, immature B and mature recirculating B cells in bone marrow. **(E)** Expression of TLR4 in the indicated B lineage populations. The mean + SD population frequencies in total bone marrow are indicated in panels D and E. Five 5 month old female B6 mice were analyzed individually and a representative plot is shown.

**Supporting Table 1** Antibodies, clone numbers used in this study and sources.

| **Antibodies for immunostaining (Flow Cytometry)** | **SOURCE** | **Cat #** |
| --- | --- | --- |
| Purified CD16/32 (Clone: 93) | ThermoFisher Scientific | 14-0161-86 |
| Rat anti-mouseCD93- FITC (Clone: AA4.1) | ThermoFisher Scientific | 11-5892-82 |
| Goat (Fab’)2 anti -mouse IgM- PE (polyclonal) | SouthernBiotech | 1020-09 |
| Rat anti-mouse CD45R (B220)- PE-Cy5.5 (Clone: RA3-6B2) | ThermoFisher Scientific | **35-0452-82** |
| Rat Anti-Mouse CD24- PE-Cy™7 (Clone: M1/69) | BD Biosciences | 560536 |
| Rat Anti-Mouse CD43- APC (Clone: S7) | BD Biosciences | 553159 |
| Rat Anti-Mouse CD249 (Ly-51)- biotin (Clone: 6C3)) | BD Biosciences | 553159 |
| Rat Anti-Mouse CD19-Pacific Blue (Clone: 1D3) | ebiosciences | 48-0193-82 |
| Rat Anti-Mouse IgD-BV605™ (clone: 11-262c.2a) | Biolegend | 405727 |
| Streptavidin-APC-Alexa Fluor™ 750 | ThermoFisher Scientific | SA1027 |
| Biotin- Mouse Anti-Mouse CD284 (TLR4, clone UT41) | ThermoFisher Scientific | 13-9041-80 |
| **Antibodies for immunostaining (Imaging)** | **SOURCE** | **Cat #** |
| Endomucin Polyclonal Antibody | ThermoFisher Scientific | PA5-47648 |
| Anti-mouse CD19 (Clone: ebio103(1103)) | ThermoFisher Scientific | 14-0193-85 |
| Donkey Anti-rat IgG (H+L) AlexaFluor 555 | ThermoFisher Scientific | A48270 |
| AlexaFluor 647 Donkey Anti-goat IgG (H+L) | ThermoFisher Scientific | A21447 |
| Rat IgG anti- Mouse CD31 Antibody (clone: 390) | BD Biosciences | 553708 |
